# Surface‐Engineered Dual‐Emission of Carbon Dots for Ratiometric Detection of Hydroxyl Radicals

**DOI:** 10.1002/nap2.70116

**Published:** 2026-05-01

**Authors:** Yatao Pan, Shihong Wang, Wenjing Zhang, Jing Ning, Jingchun Yang, Xia Ran, Lijun Guo

**Affiliations:** ^1^ Henan Province Engineering Research Center of Optoelectronic Technology and Advanced Manufacturing, and School of Physics Henan Normal University Xinxiang China; ^2^ School of Physics and Electronics, Henan Key Laboratory of High Efficiency Energy Conversion Science and Technology Henan University Kaifeng China

**Keywords:** carbon dots, dual emission, photophysics, ratiometric sensing, surface states

## Abstract

Carbon dots (CDs) have emerged as versatile photoluminescent nanomaterials with exceptional optical properties and biocompatibility, demonstrating considerable potential in bioimaging, energy conversion, and sensing applications. However, the development of aqueous CDs with tunable red dual‐emission remains challenging owing to limitations in electronic energy‐level alignment and ongoing debates concerning the photoluminescence mechanisms. Herein, we report a facile hydrothermal synthesis strategy employing trehalose as the carbon precursor and sulfuric acid serving as both an oxidation regulator and a polymerization agent to regulate the distribution of surface states and endow the CDs with dual emission properties. The resulting CDs exhibit well‐resolved tunable dual emission bands around 450 and 590 nm, attributed to core states and surface‐oxidized states, respectively. Time‐resolved spectroscopic investigations reveal that the engineered surface motifs effectively facilitate ultrafast electron migration from the carbon core to the oxidized states. Intriguingly, hydroxyl radicals induce selective static quenching of the surface‐oxidation‐associated red emission, endowing CDs‐based sensors with ultralow detection limits, self‐calibration capabilities, and real‐time visualization through ratiometric performance. Furthermore, the as‐prepared unique CDs have been validated for the reliable quantification of hydroxyl radical levels in plant onion cells under oxidative stress.

## Graphical abstract

Oxidation‐engineered CDs with tunable dual emission (450/590 nm) exhibit oxidation state‐driven electron redistribution and core‐to‐surface transfer within 5 ps, achieving 59 nM ·OH detection via ratiometric sensing and enabling dual‐channel ·OH monitoring in onion cells.

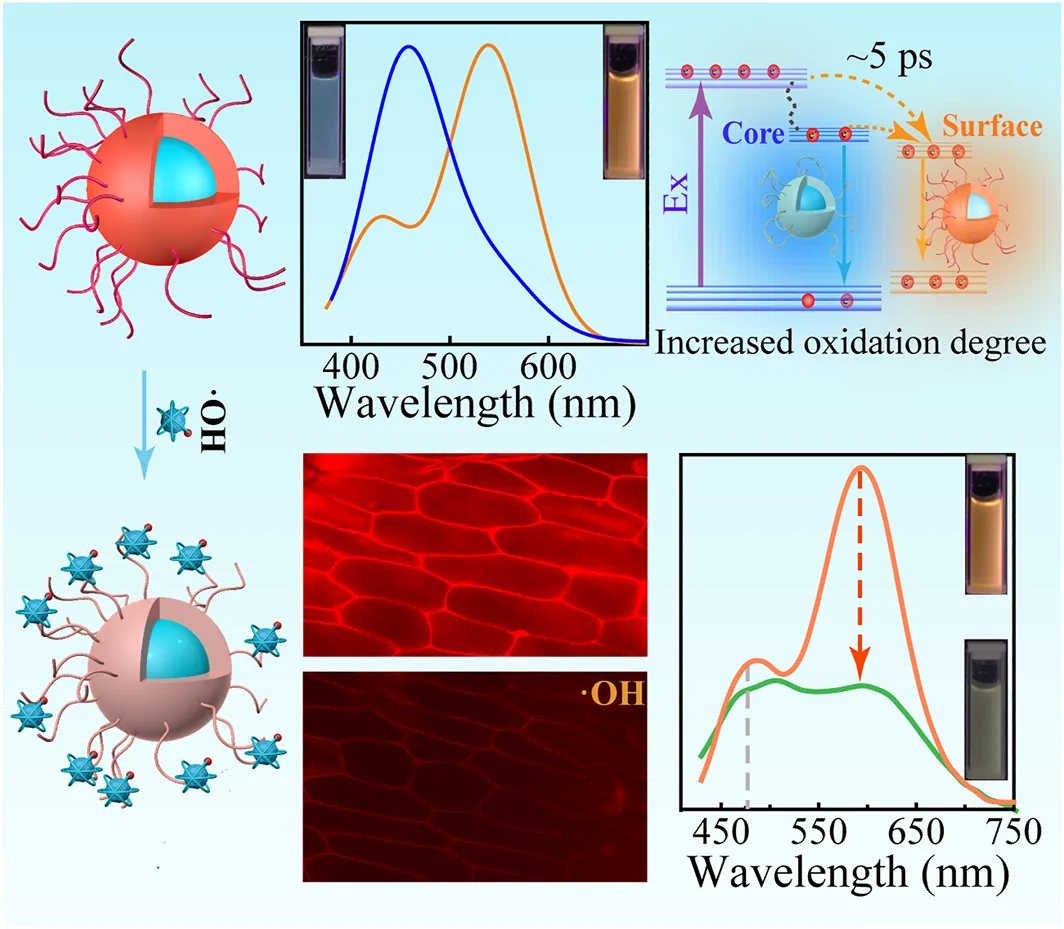

## 1 Introduction

As an emerging class of carbon nanomaterials, carbon dots (CDs) have garnered significant research interest over the past decade due to their distinctive characteristics, including exceptional biocompatibility [1], tunable bandgaps [2], superior photoluminescent properties [3, 4, 5], facile surface functionalization [6], and minimal cytotoxicity [7]. These unique properties have enabled substantial deployment across multiple domains, encompassing laser technologies [8, 9], displays engineering [10], catalytic systems [11, 12], information encryption [13], sensing platforms [14], bioimaging agents [15], energy harvesting [16], photodynamic/photothermal therapies [17, 18], photodetection devices [19, 20], and so on. Notably, the photoluminescence (PL) of CDs can be effectively modulated through synergistic strategies, including quantum confinement via sp^2^ domain size control [21], heteroatom‐mediated [22] electronic tailoring, and surface state modulation through covalent/noncovalent functionalization [23]. However, most of the currently reported CDs exhibit emission spectra confined to the blue‐green region (< 550 nm), which is primarily attributed to undersized sp^2^ domains and conjugated trap states that fail to activate the red‐shifted fluorescence, also severely constraining full‐spectrum tunability across the visible‐NIR range. Furthermore, current synthetic approaches remain inadequate in controlling reaction kinetics, which constrains targeted modulation of electronic energy states, while persisting controversies regarding emission mechanisms further constrain rational design in CDs. Consequently, developing multicolor‐emitting CD materials and elucidating their underlying photophysical mechanisms represents a critical research imperative.

The diverse surface moiety of CDs provides discrete coordination sites for analyte recognition. The interaction with target species typically induces perturbations in the electronic energy structure or the surrounding dielectric environment of CDs, substantially inducing alterations in the optical properties of the CDs, such as the PL quenching/enhancement or emission peak wavelength shifts [24], which form the basis for quantitative detection. However, conventional quantification optical strategies often depend on PL intensity changes at a single emission wavelength, which suffer from significant limitations due to the inherent vulnerabilities to various interferences, such as instrument setting fluctuation, probe microenvironment changes, and photobleaching effects, all of which can distort the PL signal and compromise measurement reliability. To address these intrinsic challenges, dual‐emissive CDs have emerged as promising alternatives owing to their unique optical properties, which originate from the adjustable energy‐level engineering of the electronic states via control molecule [25], surface, and core states. Additionally, dual‐emissive CDs can also serve as an ideal foundation for self‐calibrating ratio‐metric multicolor optical probes, broadening their applications in advanced sensing and even the next‐generation optoelectronic devices [26]. For example, Kamel et al. synthesized dual‐emissive nitrogen‐doped CDs via microwave‐assisted pyrolysis of sugarcane bagasse and citric acid precursors [27], exhibiting characteristic PL bands at 442 nm from surface C=O/C=N transitions and 511 nm arising from oxygen vacancy defect recombination. Zhang et al. prepared CDs possessing blue and green emissions through hydrothermal treatment of sodium citrate, triethylenetetramine, and rose bengal, with the blue emission centered from amide‐bond‐related surface states and the green emission arising from halide‐lost rose bengal‐derived fluorophores [28]. Irshad et al. synthesized dual‐emissive CDs via solvothermal treatment of o‐phenylenediamine and lignin, with peaks at 463 and 557 nm originating from surface‐defect states associated with interfacial dangling bonds [29]. Although significant advances have been made in energy‐level engineering of dual‐emissive CDs, critical challenges persist regarding sustainable development and effective optical bandgap modulation. Current synthesis methods mainly use multi‐component chemical precursors instead of eco‐friendly biomass sources, thereby limiting their green application potential. Additionally, existing dual‐emissive CDs typically demonstrate static excited‐state electron populations that lack facile external modulation strategies, and the fundamental photophysical mechanisms underlying their dynamic interplay between distinct state transitions still remain debated [30]. Therefore, comprehensive studies are needed to unravel these structure–property relationships, paving the way for rationally designed tunable dual‐emissive systems with tailored performance characteristics.

In this study, we engineered dual‐emissive CDs through a facile method, focusing on the multitunable PL properties by modulating surface oxidation states to achieve effective control over photophysical behavior; the trehalose was selected as a precursor for its dual capacity to stabilize molecules under dehydration stress and scavenge radicals directly under thermal extreme conditions [31, 32, 33]. Notably, these dual‐emissive CDs exhibited surface oxidation‐tunable excited‐state electron redistribution that simultaneously enables ultrafast (< 5 ps) charge transfer from the carbon core to surface states. Interestingly, the red emission centered at around 590 nm demonstrated a specific and selective response to hydroxyl radicals (·OH). In contrast, the blue‐band emission near 450 nm served as an intrinsic reference signal to enhance detection precision and sensitivity. By tracking ·OH levels through PL intensity ratio analysis, we developed an innovative CDs‐based ratiometric sensing platform. The intrinsic dual‐emission capability of this nano system further allowed in situ visualization of exogenous reactive oxygen species dynamics within plant cells, leveraging its self‐calibrating spectral characteristics for enhanced measurement reliability.

## 2 Experimental Section

### 2.1 Materials

Trehalose, glutathione (GSH), ferrous sulfate (FeSO_4_·7H_2_O,≥ 99.0%), sodium borohydride (NaBH_4_), 3,3′,5,5′‐Tetramethylbenzidine (TMB), sodium hypochlorite (NaClO), terephthalate dianion (TA), 2‐hydroxyterephthalate (HTA), xanthine, xanthine oxidase, and hydrogen peroxide (H_2_O_2_, 30% w/w) were obtained from Shanghai Aladdin Biochemical Technology Co. Ltd. Sulfuric acid was supplied by Luoyang Haohua Chemical Co. Ltd. To ensure experimental consistency and accuracy, ultrapure water (Milli‐Q system) was employed in all protocols, whereas all chemical reagents were employed directly from the supplier without further treatment.

### 2.2 Preparation of Multi‐Color Dual‐Emission CDs

Trehalose (200 mg) was dissolved in ultrapure water with varying volumes of sulfuric acid (0, 10, 40, 60, or 150 μL). Subsequently, the total volume was adjusted to 10 mL by adding ultrapure water. After vigorous stirring until complete dissolution, the homogeneous solution was sealed in a polytetrafluoroethylene‐lined steel autoclave and heated at 160°C for 8 h. Following natural cooling to ambient temperature, the resulting colloidal suspension was centrifuged at 10,000 rpm for 8 min. The upper liquid phase was subsequently collected and kept at 4°C for further characterization. Based on systematic variations in sulfuric acid concentration, the resulting CDs were labeled as B‐CDs (0 μL), G‐CDs (10 μL), W‐CDs (40 μL), Y‐CDs (60 μL), and O‐CDs (150 μL), where the nomenclature reflected their distinct bathochromic shifts in PL emission correlated with increasing acid concentration.

### 2.3 Characterizations

Optical characterization was performed on a Cary 5000 UV–Vis–NIR spectrophotometer (Agilent) and a PerkinElmer LS‐55 fluorescence spectrometer. Chemical functional groups and elemental composition were probed via Fourier transform infrared (FT‐IR) spectroscopy on a Bruker VERTEX 70 spectrometer and X‐ray photoelectron spectroscopy (XPS) using a Thermo Scientific ESCALAB 250 Xi system equipped with a monochromatic Al Kα source. For morphological evaluation, atomic force microscopy (AFM) samples were prepared by depositing 15 μL of CDs dispersion onto freshly cleaved mica, incubating for 2 min, rinsing with ultrapure water, and nitrogen‐drying before imaging with an NT‐MDT Solver P47H system in tapping mode (resonant frequency: 320 kHz). The microstructure of the O–CDs was characterized by high‐resolution transmission electron microscopy (HR‐TEM) using a JEM‐2100 instrument (JEOL, Japan).

### 2.4 Generation and Quantification of ·OH

·OH were generated via the Fenton reaction, wherein H_2_O_2_ was added in excess to account for side reactions and radical consumption. The ·OH yield was quantified using TA as a fluorescent probe, which selectively reacts with ·OH to produce highly fluorescent HTA, and the concentration was then determined by measuring the resultant fluorescence intensity relative to a standard curve. For the generation of reactive oxygen species, ClO^−^ was sourced directly from NaClO solution. Singlet oxygen (^1^O_2_) was generated chemically through the reaction of NaClO with H_2_O_2_ under acidic conditions, whereas the superoxide anion radical (·O_2_^−^) was produced enzymatically by the xanthine oxidase‐catalyzed oxidation of xanthine.

### 2.5 Ratiometric Detection of ·OH

1.0 mg of O‐CDs powder was dispersed in 2.5 mL ultrapure water via ultrasonication. To evaluate selectivity, 100 μL aliquots of 30 μM solutions containing various analytes (Na^+^, K^+^, Mg^2+^, Ca^2+^, Cu^2+^, Zn^2+^, Fe^2+^, Fe^3+^, Mn^2+^, GSH, ·OH, ClO^−^, ^1^O_2_, ·O_2_^−^, and H_2_O_2_) were sequentially introduced into the O‐CDs dispersion. Then, the emission spectra were acquired using a PerkinElmer LS‐55 fluorescence spectrometer.

### 2.6 Plant Cell Imaging

Fresh onion epidermal specimens were incubated in a 20 μg mL^−1^ CDs suspension (1 × PBS, pH 7.4) for 60 min at 25°C. Subsequently, the unbound CDs were removed via three sequential washes with ultrapure water, followed by mounting on the glass petri dish. Graded concentrations of exogenous ·OH were added to the culture medium for 15 min; after that, fluorescence imaging was conducted using a Nikon AIR MP+ confocal laser scanning microscope equipped with a 25 × water‐immersion objective. Samples were photoexcited at 405 nm (diode laser), and emission signals were collected through dual‐bandpass filters: green and red channels.

## 3 Results and Discussion

The tunable dual‐emissive CDs were prepared through a facile strategy using trehalose as carbon/oxygen precursors and concentrated sulfuric acid as both carbonization catalyst and surface oxidation modulator. To elucidate how sulfuric acid concentration modulates the photophysical properties of CDs, we conducted a comparative analysis of morphology and surface moieties across different samples. The morphological features of five distinct CDs were investigated through AFM. Figure 1a–f revealed that all CDs exhibited monodisperse spherical morphologies, with average diameters of 2.9 nm, 4.1, 5.5, and 7.8 nm. This size modification not only confirmed acid‐mediated regulation of CDs dimensions but also established size‐dependent quantum confinement effects as a potential determinant of their optical properties.

**FIGURE 1:**
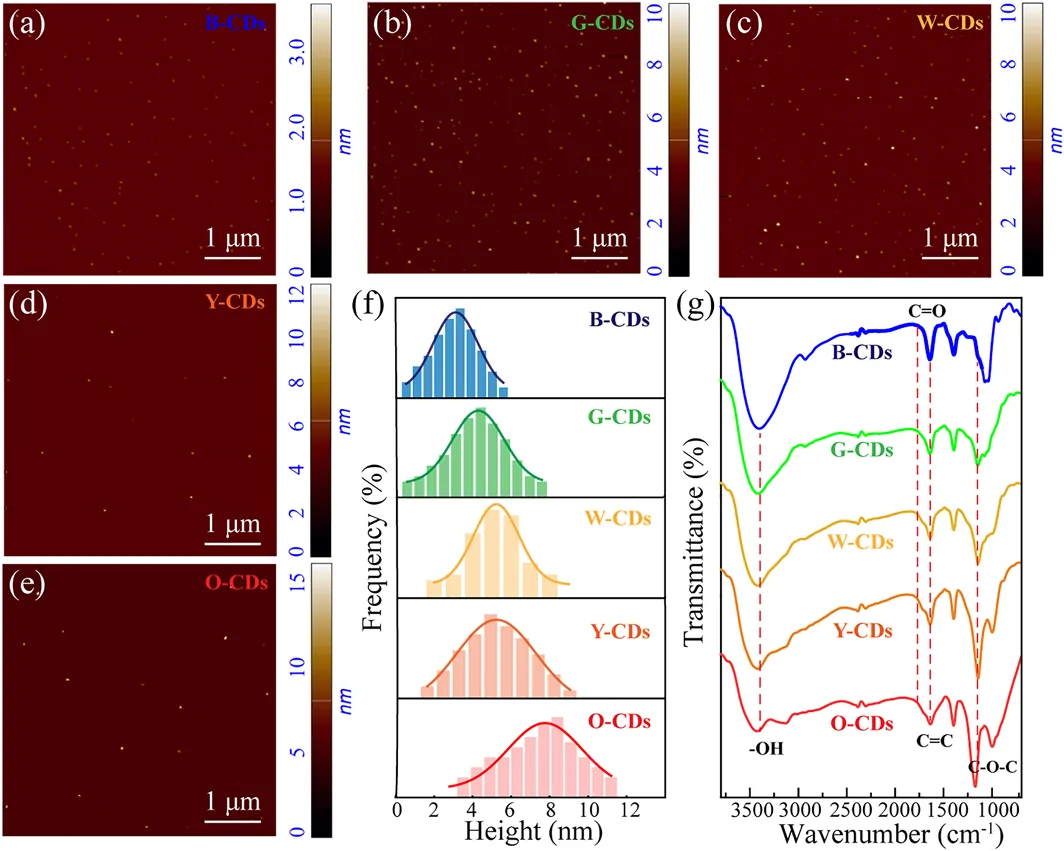
(a–e) AFM images of CDs prepared with different concentrations of H_2_SO_4_. Diameter histograms (f) and FT‐IR spectra (g) of different types of CDs.

To elucidate the acid‐mediated carbonization mechanism and monitor bonding transformations, FTIR analysis was conducted on the synthesized samples, with specific emphasis on the evolution of oxygen‐ and carbon‐containing functional groups.

As shown in Figure 1g, all synthesized samples exhibited distinct FTIR absorption spectra corresponding to different functional groups: a wide O–H stretching band centered near 3450 cm^−1^, carbonyl stretching modes at 1700 cm^−1^, aromatic C=C skeletal vibrations around 1590 cm^−1^, and C–O stretching modes near 1165 cm^−1^, concurrently confirming the oxygen‐rich surface moieties of the CDs. The gradual attenuation and eventual disappearance of C–H vibrational modes (2882 cm^−1^), originating from nonconjugated trehalose precursors, confirmed dehydration and condensation as dominant reaction pathways during hydrothermal synthesis. Comprehensive spectral evolution analysis revealed an inverse relationship between acid concentration and conjugation integrity, where concentrated acid conditions intensified oxidative functionalization as evidenced by rising C=O and C–O–C content and broadening of C=C vibrational bands. These structural modifications demonstrated a modulated transformation from B–CDs to O–CDs, with enhanced surface oxidation potentially regulating PL of CDs through oxidative state manipulation.

XPS analysis was further used to verify the chemical states and elemental distribution within the CDs. Survey spectra (Figure 2a,b) exclusively exhibited characteristic C 1s (284.6 eV) and O 1s (531.6 eV) signals, and quantitative analysis of the C/O atomic ratio revealed a strong correlation between oxygen degree increment and progressive emission bathochromic shifts from B–CDs to O–CDs [34, 35]. Deconvolution of fine‐scan O 1s spectra confirmed oxygen speciation into C–O (533.6 eV) and C=O (531.7 eV) moieties (Figure 2c,d), whereas corresponding C 1s analysis identified three distinct carbon‐bonding configurations (Figure 2e,f): graphitic C=C (284.2 eV), hydroxyl/ether C‐O (286.2 eV), and carbonyl C=O (288.4 eV). Quantitative evolution of these moieties demonstrated a systematic increase in surface reconstruction C=O species from 34% in B–CDs to 43% in O–CDs (Supporting Information S1: Table S1), with corresponding C 1s analysis confirming this oxidation shift via attenuated C=C and intensified C=O contributions (Supporting Information S1: Table S2). This coordinated transformation validated a dehydration‐driven surface modification mechanism during hydrothermal processing. XPS analysis revealed no detectable S 2p signal, indicating negligible sulfur doping into the carbon framework. Furthermore, progressive oxygen enrichment directly correlated with bathochromic emission shifts, evidencing oxygen‐mediated surface reconstruction via hydrothermal dehydration that tailors electronic structures through π‐conjugation disruption and mid‐gap state induction. The relationship between surface oxidation states and PL characteristics in CDs has been well‐established as evidenced by oxidative modification generating defect‐centered recombination sites [36] or inducing localized structural distortions that modify electronic transitions [37], with these modifications potentially altering the absorption behavior and contributing to red‐shifted emission [38, 39, 40].

**FIGURE 2:**
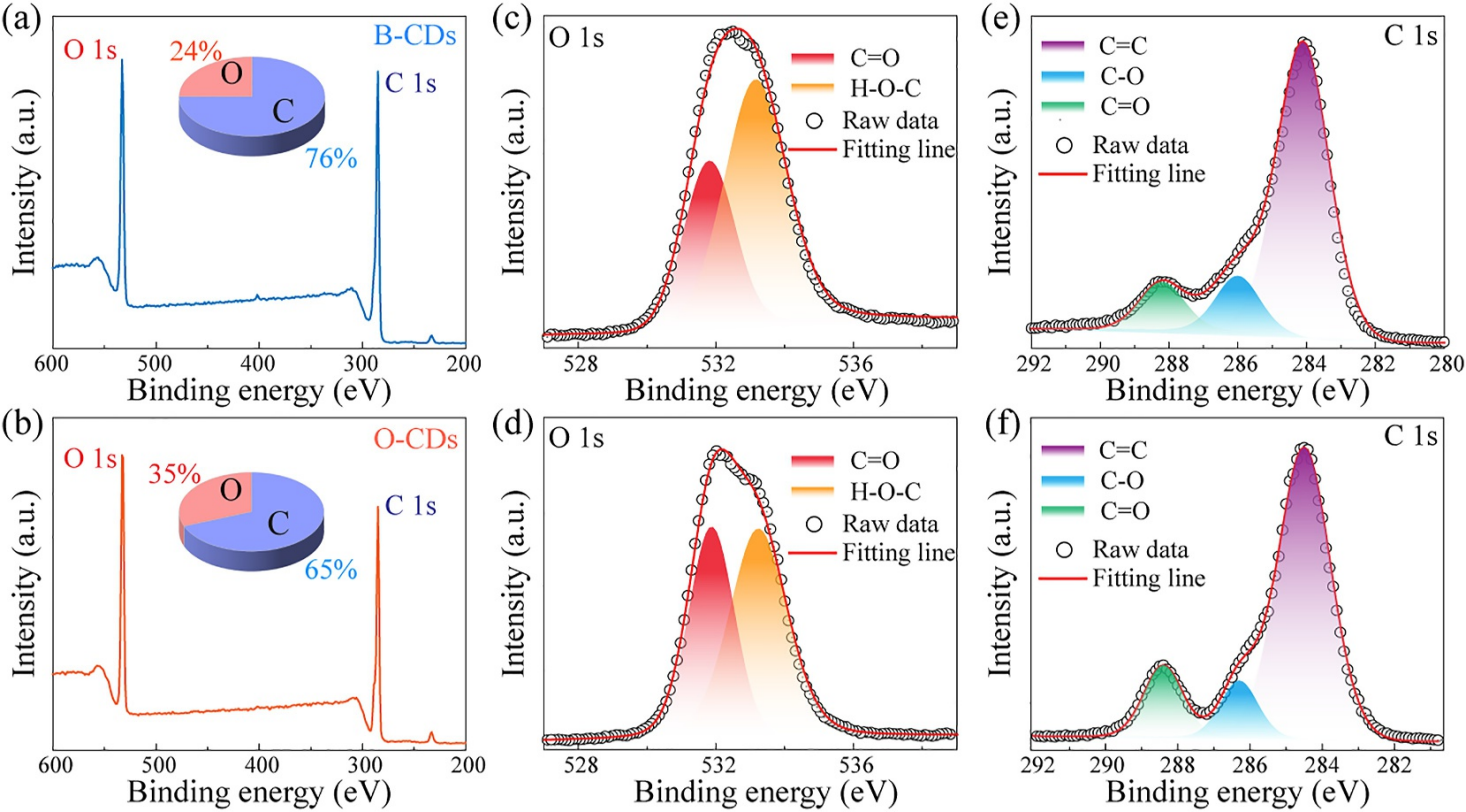
XPS full‐range survey scans of B–CDs (a) and O–CDs (b). High‐resolution O 1s spectra of B–CDs (c) and O–CDs (d). Chemical bonding configurations of C 1s spectra (e, f).

Furthermore, we conducted a systematic investigation into the photophysical properties of the series of CDs with particular emphasis on elucidating the synergistic effects of core graphitization and surface functionalization that modulate their optical behaviors. Figure 3a–d displayed the optical spectra of the CDs, where the UV‐vis absorption of B–CDs (Figure 3a) showed an intense absorption band around 284 nm and a very weak tail state. Meanwhile, the shoulder band around 350 nm became increasingly pronounced from G–CDs to O–CDs, a trend that aligned with the enhanced oxidation state observed in the FTIR and XPS analysis. The absorption band around 284 nm originated from π→π* transitions within sp^2^‐hybridized carbon networks [41], whereas the 350 nm feature arose from n→π* transitions involving surface oxygen functionalities [8, 42]. Notably, as the sulfuric acid concentration increased, the absorption bands attributed to the sp^2^‐hybridized carbon cores underwent a progressive broadening and intensity attenuation, indicative of structural disordering or lattice strain. Concurrently, lower‐energy tail state 300–450 nm region electronic transitions became intensified, suggesting the emergence of new electronic states or surface‐functionalized moieties that participated in charge‐transfer processes.

**FIGURE 3:**
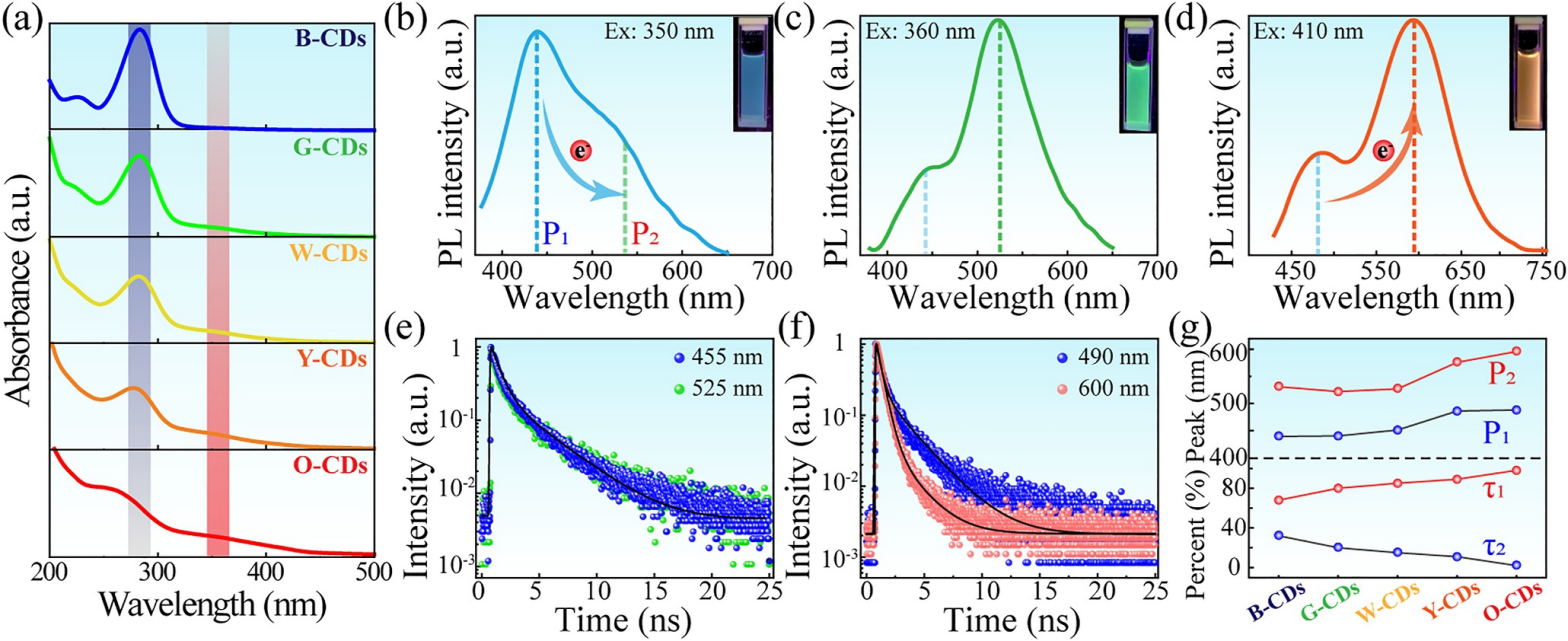
(a) Absorption profiles of the five samples. PL spectra of B–CDs (b), G–CDs (c), and O–CDs (d), with the corresponding excitation wavelengths and photographs of solutions under UV light shown in the inset. PL lifetime decay profiles of B–CDs (e) and O–CDs (f) measured at different detection wavelengths under 365 nm excitation. (g) Relative PL intensity and lifetime of core and surface states across the five CDs.

The excitation‐emission spectra of the prepared samples were presented in Figure 3b–d and Supporting Information S1: Figures S1 and S2. All samples exhibited two distinct emission bands: a short‐wavelength band and a progressively dominant red‐band emission. The intensity ratio of the red‐band emission increased from 0.56 to 2.3, accompanied by a red shift of 55 nm. PL spectra recorded at different wavelengths revealed that B–CDs exhibited slight excitation‐dependent behavior, whereas G–CDs and O–CDs showed negligible excitation dependence, suggesting that the red‐band emission likely originated from surface‐oxygen states [43], whereas the short‐wavelength emission arose from core‐state electronic transitions. Furthermore, the observed excitation wavelength redshifts correlated with enhanced absorption contributions in the tail‐state region, which aligned with the increase of surface defect density as corroborated by XPS analysis detecting elevated oxygenated groups. Treatment of both G–CDs and O–CDs with NaBH_4_ resulted in a progressive blue shift in emission spectra, along with a notable enhancement in the short‐wavelength band intensity (Supporting Information S1: Figure S3). As the amount of reducing agent increased, the proportion of blue‐band emission rose in both samples, suggesting a reduction in surface‐oxidized groups and an increase in the conjugation degree of the carbon core, thereby amplifying the contribution of core‐state emission. This spectral evolution demonstrated that oxygen group removal attenuated defect‐mediated red emission, thereby confirming the origin of red‐band emission in oxygen‐derived surface defects. Therefore, the diverse PL behaviors of CDs could be attributed to synergistic interactions between the sp^2^‐hybridized carbon matrix and peripheral surface states rather than to the quantum confinement effect. Additionally, in contrast to conventional CDs that exhibited concentration‐dependent emission shifts, both G–CDs and O–CDs maintained constant PL spectra across varying concentrations (Supporting Information S1: Figure S4). AFM and TEM images (Supporting Information S1: Figure S5) further confirmed the absence of aggregation in CDs, concurrently demonstrating that their PL was governed by intrinsic engineered centers within individual CDs. PL decay curves (Figure 3e,f, Supporting Information S1: Figure S6) were analyzed to investigate carrier dynamics and origins of radiative centers between the samples. All curves could be biexponentially fitted, confirming two distinct emission centers within the CDs. The excited‐state lifetime exhibited a progressive shortening from 3.49 ns (B–CDs) to 0.71 ns (O–CDs), accompanied by a concomitant increase in the short‐lived component (τ_1_) contribution from 68% in B–CDs to 96% in O–CDs (Figure 3g, Supporting Information S1: Table S3). These lifetime and spectroscopic changes could be attributed to surface‐localized n→π* excitations in oxygen‐derived groups (C=O/C‐O) as verified by XPS O 1s analysis. The long‐lived component (τ_2_), between 2.1 and 4.5 ns, corresponds to π–π* transitions in sp^2^‐hybridized carbon cores. Notably, O–CDs exhibited the highest proportion of the short‐lived component at 96%, correlating with the elevated surface oxidation degree indicated by the XPS O/C ratio of 0.54 compared to 0.32 for B–CDs. The synergistic interaction between the dual emission centers drove the observed lifetime retraction and emission wavelength red‐shift, demonstrating defect‐mediated modulation of exciton recombination pathways through controlled surface oxidation.

Although quantum confinement theory predicted size‐dependent emission wavelengths in CDs [44] and time‐dependent density functional theory (TD–DFT) calculations suggested 999.5 nm emission for 2.3 nm zigzag‐edged CDs [45], synthesized CDs exceeding this size exhibited pronouncedly shorter wavelengths relative to theoretical predictions. This deviation suggested sp^3^‐hybridized carbon and functional groups fragment the sp^2^‐conjugated networks to modulate the energy levels. Additionally, HR–TEM characterization of O–CDs (Supporting Information S1: Figure S5) revealed that only a minority of particles exhibited clear lattice fringes, whereas the majority lacked large‐area sp^2^‐hybridized inner structures. Consequently, we inferred that quantum confinement contributed minimally to the emission properties, and the observed spectral changes arose primarily from oxygen‐doping‐induced modifications to the electronic structure. The newly formed electron‐withdrawing C=O and C–O moieties restructured defect distributions and established intermediate states between π and π* orbitals, comprehensively transforming the optoelectronic properties [37], which was directly evidenced by the sulfuric acid concentration‐dependent emission variations shown in Figure 3d.

To further resolve the photoinduced charge carrier dynamics and oxidation‐mediated charge redistribution in CDs systems, we employed ultrafast transient absorption spectroscopy with 350 nm excitation. Figure 4a,b showed the 3D TA maps of B–CDs and O–CDs, covering the spectral range of 355–660 nm across varying time delays. The observed positive differential absorption signals originated from multiple excited‐state absorption (ESA) transitions, whereas negative ΔOD features corresponded to either ground‐state bleaching (GSB) or stimulated emission (SE). Through careful comparison with steady‐state absorption spectra and considering the minimal absorption cross‐section of O–CDs near 625 nm, we conclusively assigned the 625 nm feature to the SE process. Remarkably, surface oxidation not only triggered charge redistribution but also substantially modified the excited‐state relaxation pathways within these CDs systems. As shown in Figure 4c,d, the time evolution of TA spectra revealed distinct kinetic behaviors at characteristic wavelengths. The SE signal around 625 nm exhibited a gradual intensity increase during the first 30 ps (Figure 4e,f), indicative of population transfer from higher‐level excited states with a growth time constant of 5.5 ps (Supporting Information S1: Table S4). Concurrently, the emergence of ESA around 490 nm within 10 ps indicated efficient energy transfer from other excited states, as these phenomena were absent in pristine B–CDs. This contrast highlighted the impact of surface oxidation on the excited‐state dynamics of CDs, which enhanced the absorption cross‐section of tail states in O–CDs and facilitated excited‐state electron migration and population transfer from core to surface states (Figure 4g). This modification then led to pronounced enhancement of surface‐mediated red emission, which was beneficial for highly specific ·OH detection and monitoring.

**FIGURE 4:**
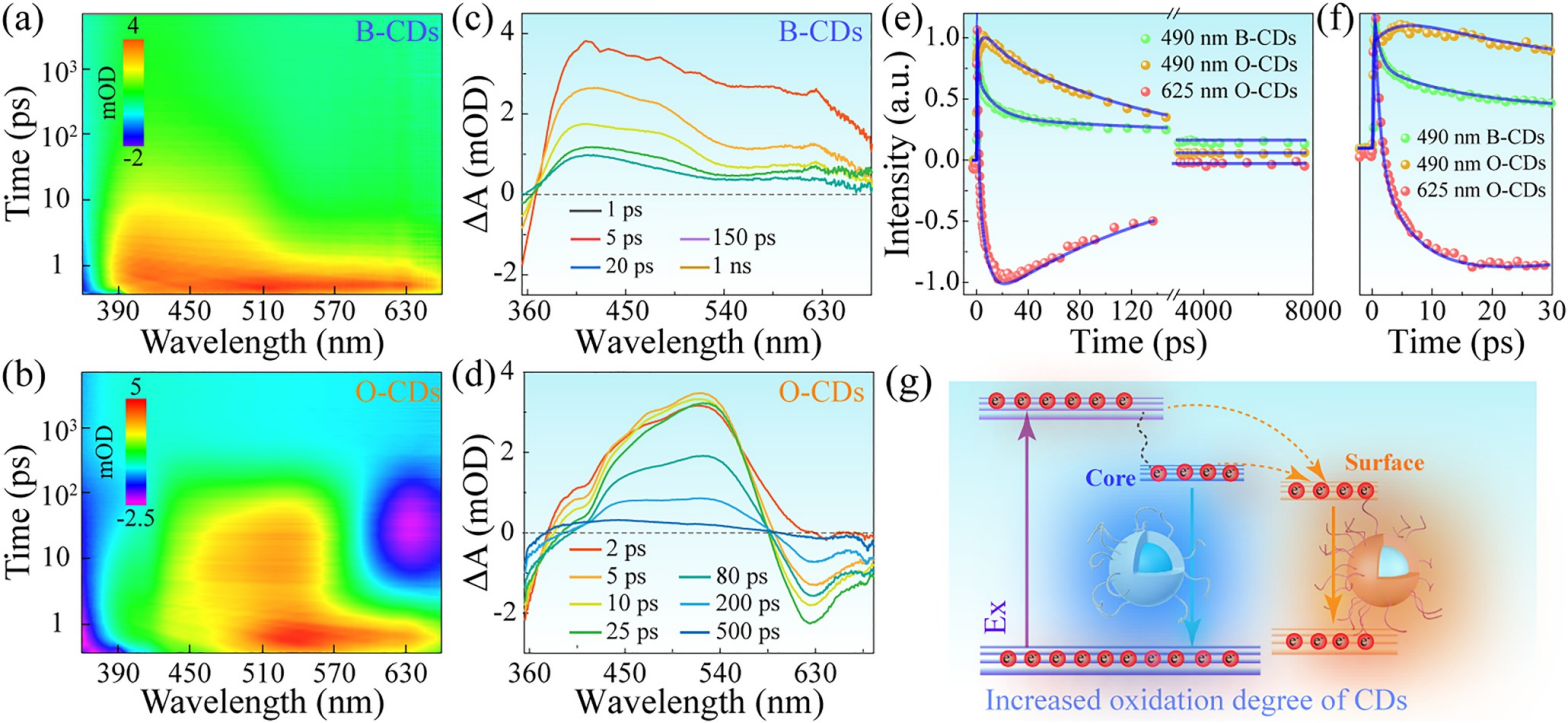
Evolution of ΔOD captured in the femtosecond transient absorption maps of B–CDs (a) and O–CDs (b) under 350 nm excitation, plotted against probe wavelength and time delay. Time‐resolved differential spectra revealing distinct excited‐state dynamics for B–CDs (c) and O–CDs (d). (e) Kinetics profiles at selected wavelengths with corresponding fits for B–CDs and O–CDs. (f) Expanded view of initial relaxation processes (0–30 ps). (g) Proposed relaxation mechanism illustrating oxidation‐dependent excited‐state dynamics in CDs.

Extensive research has established that trehalose provides exceptional protection to biological systems through two primary mechanisms: biomacromolecule stabilization under extreme environmental conditions and hydroxyl‐mediated radical scavenging via redox‐neutralizing pathways [46]. Inspired by these findings, we proposed to evaluate the radical scavenging capacity of CDs by harnessing the synergistic integration between the radical‐neutralizing hydroxyl moieties present in trehalose and the electron transfer conjugation domains within CDs. To assess the selectivity of O–CDs for ·OH detection in biological systems, potential interferences from common metal ions, biomolecules, and ROS were systematically evaluated. PL responses were monitored against biologically relevant cations (Na^+^, K^+^, Mg^2+^, Ca^2+^, Cu^2+^, Zn^2+^, Fe^2+^, Fe^3+^, and Mn^2+^), GSH, and ROS species (H_2_O_2_, ·OH, ClO^−^, ^1^O_2_, and ·O_2_^−^), which exhibited excellent discrimination against interferents, characterized by minimal signal variation below 15% (Figure 5a). In contrast, the introduction of ·OH resulted in significant fluorescence quenching, reducing the emission intensity to approximately 85% of its original value, thereby confirming the high selectivity of the O‐CDs for the target ·OH. Meanwhile, initial ·OH production was prepared through Fenton chemistry (Fe^2+^+H_2_O_2_), with the resultant oxidative conversion of TMB serving as the colorimetric indicator. It was worth noting that increasing O–CDs concentration caused a progressive decrease of oxTMB's characteristic absorbance at 650 nm (Supporting Information S1: Figure S7), demonstrating the potent ·OH scavenging capacity of O‐CDs. Although both B–CDs and O–CDs could scavenge ·OH radicals (Supporting Information S1: Figures S8 and S9), the O–CDs exhibited enhanced trapping efficiency (ΔA/A_0_ = 0.39 at 1.5 mg/mL) relative to B–CDs (ΔA/A_0_ = 0.15), which was attributed to their higher oxidation degree and enriched surface‐active sites, hydroxyl groups, and carbonyl moieties [41, 42]. Remarkably, O–CDs with dual emission bands exhibited different quenching responses to ·OH. The red band emission around 590 nm showed pronounced intensity attenuation (Figure 5b), which was possibly due to the presence of multiple binding sites facilitating the formation of complexes, whereas the blue band emission was less affected by radicals, suggesting that conjugated sp^2^ domains exhibited intrinsic shielding against external penetration. These considerations prompted the engineering of a dual‐emissive nano‐sensor for ·OH quantification, whose self‐referencing I_590_/I_470_ ratio inherently compensates for environmental fluctuations to afford superior accuracy over single‐channel detection. Based on the CDs‐based probing strategy, a highly selective and convenient method for ·OH determination was established. As demonstrated in Figure 5c, the ratiometric signal showed linearity within the 0–5 μM ·OH concentration range, with a calculated detection limit of 59 nM, comparable to those of other CDs‐based [47] or nanoparticles‐based sensors for ·OH [48, 49, 50, 51] (Supporting Information S1: Table S5). It was noteworthy that the rationally designed CDs, which featured effectively tunable dual‐emission properties that were intrinsic rather than reliant on external molecular or nanocrystal hybridization, not only enabled ·OH scavenging but also provided a versatile platform for developing detection devices. The results demonstrate that the O‐CDs possess outstanding selectivity and great potential for practical application in complex sample matrices. The PL decay results (Figure 5d) demonstrated negligible lifetime reduction in O–CDs upon ·OH exposure, indicating dominant static quenching where the radical formed nonfluorescent complexes without altering excited‐state dynamics. This assessment was further substantiated by the temperature‐dependent quenching constants (Supporting Information S1: Figure S10), which decrease from 1.5 × 10^5^ to 1.0 × 10^5^ L/mol as the test temperature increased from 25°C to 60°C. Before biological applications, comprehensive characterization confirmed that the O–CDs possessed exceptional stability with less than 10% PL intensity loss at 1 M ionic strength and 91% intensity retention after 10 min light exposure (Supporting Information S1: Figure S11), along with excellent biocompatibility exhibiting > 92% cell viability at 1–100 μg mL^−1^ concentrations over 24 h (Supporting Information S1: Figure S12), demonstrating their suitability as reliable probes for cellular imaging, supporting the biosafety for subsequent cellular imaging applications. Given the well‐established impairment of chloroplast and mitochondrial function under high‐temperature stress, which accelerates reactive oxygen species generation and induces oxidative damage in plant cells, we evaluated the thermal stability of O–CDs (Supporting Information S1: Figure S13). The results revealed that their fluorescence intensity declined gradually below 40°C with an approximate 10% reduction, whereas a more pronounced decrease occurred at higher temperatures. These findings indicated that O–CDs retained sufficient thermal stability under moderate heat stress, suggesting that their thermal stability and ·OH scavenging capacity would remain effective under typical high‐temperature conditions encountered in the plant physiological environment. In cellular imaging experiments, onion cells were cultured with O–CDs for 1 h, followed by thorough washing with deionized water to remove noninternalized CDs. Subsequently, a confocal microscopy image (Figure 5e) revealed that the samples predominantly accumulated in the cell walls due to their affinity for polysaccharide and cellulose matrices in the structure, while also demonstrating their effective membrane permeability and intracellular localization, which stemmed from the compact dimensions and surface‐modified functional moieties of the nanomaterials that promoted physiological compatibility. More importantly, the characteristic dual‐emissive profile of O–CDs was preserved in the cellular environment, with clearly resolved green and red emission channels corresponding to the core and surface states. To evaluate the responsiveness of O–CDs to ·OH levels, we performed quantitative PL imaging of onion cells treated with varying concentrations of radicals under 405 nm excitation. The image data (Figure 5e,f) revealed a distinct differential response between the two channels: while the green channel exhibited only moderate PL intensity attenuation (about 20%) even at high radical concentrations, the red channel showed significantly greater sensitivity with up to 75% fluorescence quenching. This observation verified that the radical interaction primarily targeted surface moieties governing red‐band PL, whereas the core‐mediated green emission remained relatively unaffected. The consistency between these cellular observations and in vitro characterization data (Figure 5b) validated the reliability of O–CDs for both detection and potential scavenging of ·OH in biological systems.

**FIGURE 5:**
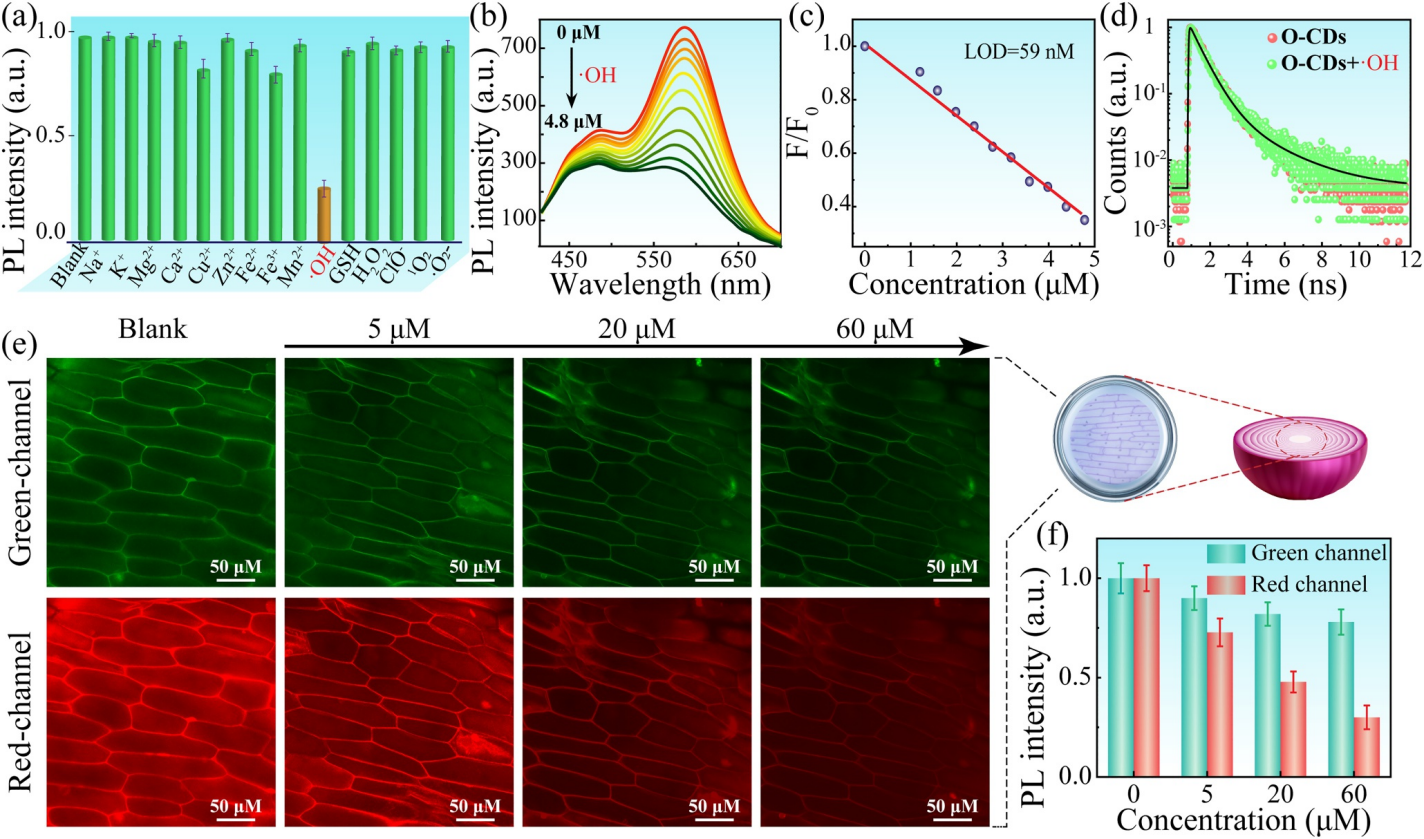
(a) Selectivity assessment of O–CDs toward ·OH versus interferents. (b) PL spectra of O–CDs recorded at varying ·OH concentrations. (c) Relative PL intensities (F/F_0_) plot against ·OH concentrations. (d) Comparison of O–CDs lifetime decay at 600 nm with/without ·OH under 365 nm excitation. (e) Dual‐channel confocal microscopy imaging of onion cells following treatment with O–CDs and subsequent exposure to varying concentrations of ·OH. (f) Quantitative comparison of dual‐channel PL intensities versus ·OH concentrations.

## 4 Conclusion

We developed a facile one‐pot synthetic strategy to engineer trehalose‐derived CDs with tunable dual‐emission properties. By adjusting acid‐mediated dehydration and condensation processes, systematic modulation of PL from blue‐emitting core states near 450 nm to red‐emitting surface‐oxidized states around 590 nm was achieved. This structural control also drove excited‐state electron redistribution and facilitated ultrafast core‐to‐surface electron transfer occurring within approximately 5.5 ps. Comprehensive spectroscopic analysis revealed that the redshift emission correlated with increasing oxygen content, which arose from the formation of electron‐withdrawing C=O/C–O groups that modified the excited‐state electron distribution. The developed ratiometric probe enabled ·OH detection with a limit of detection of 59 nM through static quenching of surface oxygen groups while maintaining stable core emission for internal calibration. The promising application in monitoring cellular ·OH fluctuations demonstrated its biological utility, with tunable dual‐emission and surface reactivity offering versatile sensing capabilities.

## Author Contributions

**Yatao Pan:** funding acquisition, writing – review and editing. **Shihong Wang:** investigation, writing – original draft. **Wenjing Zhang:** data curation, visualization, formal analysis. **Jing Ning:** methodology, validation. **Jingchun Yang:** methodology, visualization. **Xia Ran:** funding acquisition, resources. **Lijun Guo:** project administration, writing – review and editing, funding acquisition, supervision, conceptualization.

## Funding

This work was financially supported by the National Natural Science Foundation of China (Grants 22405078, 22105063, and 22373031), the Young Core Instructor Foundation of Henan Province (Grant 2021GGJS023), the Doctoral Scientific Research Start‐up Foundation (Grant 5101329170002), and the Postdoctoral Research Foundation of Henan Normal University (Grant 5101029470319).

## Ethics Statement

The authors have nothing to report.

## Consent

Informed consent was obtained from all individuals included in this study.

## Conflicts of Interest

The authors declare no conflicts of interest.

## Supporting information


Supporting Information S1

## Data Availability

The data that support the findings of this study are available from the corresponding author upon reasonable request.
