# Supplementary material for: Surface‐Engineered Dual‐Emission of Carbon Dots for Ratiometric Detection of Hydroxyl Radicals
Source: Nanophotonics. 2026 May 1;15(9):e70116. doi: 10.1002/nap2.70116 (PMC13642065; doi:10.1002/nap2.70116)
Supplement: Supplementary file 1 [file NAP2-15-e70116-s001.doc]

Surface-engineered Dual-emission of Carbon Dots for Ratiometric Detection of Hydroxyl Radicals

Yatao Pan ^a,b^, Shihong Wang ^a^, Wenjing Zhang ^b^, Jing Ning ^a^, Jingchun Yang ^b^, Xia Ran ^b,*^, Lijun Guo ^b,*^

^a^ Henan Province Engineering Research Center of Optoelectronic Technology and Advanced Manufacturing, and School of Physics, Henan Normal University, Xinxiang, 453007, China.

^b^ School of Physics and Electronics, Henan Key Laboratory of High Efficiency Energy Conversion Science and Technology, Henan University, Kaifeng 475004, China.

Corresponding authors. email addresses: ranxia@henu.edu.cn (X.R.) and juneguo@henu.edu.cn (L. Guo)

| Table S1. Chemical bond content of high-resolution O 1s of B-CDs and O-CDs. | | |
| --- | --- | --- |
|  | B-CDs | O-CDs |
| C=O | 34% | 43% |
| C-O | 66% | 57% |

| Table S2. Quantitative deconvolution of high-resolution C 1s spectra of B-CDs and O-CDs. | | |
| --- | --- | --- |
|  | B-CDs | O-CDs |
| C=C | 75% | 71% |
| C-O | 13% | 13% |
| C=O | 12% | 16% |

Figure S1. PL spectra of B-CDs (a), G-CDs (b), and O-CDs (c) across the 350-420 nm excitation range.

Figure S2. Excitation and emission spectra of B-CDs (a), G-CDs (b), and O-CDs (c) with selective wavelength irradiation (λ_ex_=350/380/400 nm). The inset displays aqueous dispersions under 365 nm UV irradiation.

Figure S3. Photographs of G-CDs (a) and O-CDs (b) aqueous solutions under UV irradiation with increasing NaBH_4_ concentrations. PL spectra of B-CDs (c) and O-CDs (d) solution under different concentrations of NaHB_4_. Dependence of the relative PL intensity of B-CDs (e) and O-CDs (f) on NaBH_4_ concentration corresponding to core-state and surface-state emissions.

Figure S4. PL spectra of G-CDs (a) and O-CDs (b) at varying concentrations.

Figure S5. HR-TEM characterization of the O-CDs.

Figure S6. Time-resolved PL decay profiles and bi-exponential fitting results (red lines) of G-CDs (a), W-CDs (b), Y-CDs (c) and O-CDs (d) under 365 nm excitation.

| Table S3. PL lifetime of B-CDs, G-CDs, W-CDs, Y-CDs and O-CDs at different emission wavelengths with 365 nm excitation | | | | | | | | | |
| --- | --- | --- | --- | --- | --- | --- | --- | --- | --- |
|  | | E_m_ (nm) | τ_1_ (ns) | τ_2_ (ns) | | A_1_ (%) | | A_2_ (%) | Av. τ(ns) |
| B-CDs | 525 | | 0.8 | 4.5 | 68 | | 32 | | 3.49 |
| G-CDs | 530 | | 0.5 | 3.1 | 78 | | 22 | | 2.15 |
| W-CDs | 600 | | 0.3 | 2.3 | 94 | | 6 | | 0.96 |
| Y-CDs | 600 | | 0.3 | 2.2 | 95 | | 5 | | 0.83 |
| O-CDs | 600 | | 0.4 | 2.1 | 96 | | 4 | | 0.71 |

| Table S4. TA kinetics of B-CDs and O-CDs at different emission wavelengths with 350 nm excitation | | | | | | | | |
| --- | --- | --- | --- | --- | --- | --- | --- | --- |
|  | E_m_ (nm) | τ_g_ (ps) | τ_1_ (ps) | τ_2_ (ps) | τ_3_ (ps) | A_1_ (%) | A_2_ (%) | A_3_ (%) |
| B-CDs | 490 |  | 2.3 | 575 | 4867 | 56 | 32 | 12 |
| O-CDs | 490 | 2.3 |  | 127 | 2463 |  | 91 | 9 |
| O-CDs | 625 | 5.5 |  | 135 | 2366 |  | 96 | 4 |

Figure S7. Absorbance spectra of oxTMB under different concentrations of O-CDs; the inset shows the solution color change under daylight.

Figure S8. UV-vis spectra of 5 mM TMB solutions containing H_2_O_2_ (10 μL) and FeCl_2_ (15 μL) for different concentrations of B-CDs (a) and O-CDs (b).

Figure S9. The changes of A_0_-A/A_0_ under different concentrations of B-CDs and O-CDs.

| Table S5. Comparative performance of nanomaterial-enabled ·OH detection | | | | | |
| --- | --- | --- | --- | --- | --- |
| Probes | λ_em_(nm) | Switch | Linear range(μM) | Limit(nM) | Ref |
| R-CDs | 612 | Turn-off | 0-120 | 200 | [1] |
| A-CDs | 440 | Turn-off | 1-19 | 0.4 | [2] |
| Mn-SiQDs | 515 | Turn-off | 0.8-50 | 88.4 | [3] |
| F-CDs | 450 | Turn-on | 0-12 | 95 | [4] |
| 3-CCA | 444 | Turn-on | 0-15 | 535 | [5] |
| r-CDs | 580 | Turn-on | 0-65 | 38.4 | [6] |
| CDIA@LMWC NP | 690/720 | Ratiometric | 0-200 | 5.3 | [7] |
| CDs-CdTe | 460/594 | Ratiometric | 0-0.1 | 8 | [8] |
| TPA@CDs | 326/423 | Ratiometric | 0.5-50 | 250 | [9] |
| PCuNCs-BCuNCs | 445/652 | Ratiometric | 1.5-11.5 | 150 | [10] |
| O-CDs | 480/600 | Ratiometric | 0-5 | 59 | This work |

[1] Y. Xu, C. Wang, L. Sui, G. Ran, Q. Song, "Phosphoric Acid Densified Red Emissive Carbon Dots with A Well-Defined Structure and Narrow Band Fluorescence for Intracellular Reactive Oxygen Species Detection and Scavenging," *Journal of Materials Chemistry C* 11, no. 8 (2023): 2984-2994, <https://doi.org/10.1039/D2TC04897C>.

[2] X. Deng, M. Zhang, Y. Wang, C. Li, X. Zhang, S. Weng, Y. Li, "Carbon Dots with Selective Fluorescence Response to Hydroxyl Radical for Sensitive Detection of Bleomycin," *Spectrochimica Acta Part A: Molecular and Biomolecular Spectroscopy* 306 (2024): 123582, <https://doi.org/10.1016/j.saa.2023.123582>.

[3] Y.-C. Sun, L.-F. Pang, X.-F. Guo, H. Wang, "Synthesis of Metal Ion-Tolerant Mn-Doped Fluorescence Silicon Quantum Dots with Green Emission and Its Application for Selective Imaging of •OH in Living Cells," *Microchimica Acta* 189 (2022): 60, https://doi.org/10.1007/s00604-021-05082-8.

[4] Y. Hou, J. Lin, Y. Chen, J. He, Z. Su, Q. Zheng, X. Zhao, X. Lv, X. Tang, C. Zhou, "Flammulina Velutipes-Derived Carbon Dots for Fluorescence Detection and Imaging of Hydroxyl Radical," *Spectrochimica Acta Part A: Molecular and Biomolecular Spectroscopy* 291 (2023): 122304, <https://doi.org/10.1016/j.saa.2022.122304>.

[5] K. Wang, R. Wang, Y. Fang, C. Liu, H. Zhu, X. Rong, B. Zhu, "Exploration of A New Approach for Detection of Nitrite with Hydroxyl Radical Fluorescence Probe in Aqueous Solutions," *Talanta* 275 (2024): 126118, <https://doi.org/10.1016/j.talanta.2024.126118>.

[6] J. Ma, H. Cui, Q. Pang, C. Wang, Q. Song, "Oxidative Aggregation-Induced Signal Enhancement of Carbon Dots for Dual-Mode Hydroxyl Radical Detection and Intracellular Imaging," [*Sensors and Actuators B: Chemical*](https://www.sciencedirect.com/journal/sensors-and-actuators-b-chemical) 451 (2026): 139433, <https://doi.org/10.1016/j.snb.2026.139433>.

[7] H. Gao, L. Sun, J. Li, Q. Zhou, H. Xu, X-N. Ma, R. Li, B-Y Yu, J. Tian, "Illumination of Hydroxyl Radical in Kidney Injury and High-Throughput Screening of Natural Protectants Using A Fluorescent/Photoacoustic Probe," *Advanced Science* 10, no. 33 (2023): 2303926, [https://doi.org/10.1002/advs.202303926](https://doi.org/10.1016/j.snb.2026.139433).

[8] J. Zhou, Y. Zhang, Y. Du, R. Zhao, S. Liu, W. Li, Q. Wang, P. Yang, "C-Cdte Quantum Dots as Dual-Wavelength Fluorescence and Colorimetric Probes to Detect Hydrogen Peroxide, Hydroxyl Radicals, and Iron (II) in Serum," [*ACS Applied Nano Materials*](https://pubs.acs.org/journal/aanmf6?ref=breadcrumb) 5, no. 10 (2022): 15875-15884, <https://doi.org/10.1021/acsanm.2c04099>.

[9] H. Wang, T. Fu, M. Ai, J. Liu, "Ratiometric Fluorescence Nanoprobe Based on Carbon Dots and Terephthalic Acid for Determining Fe^2+^ in Environmental Samples," *Analytical and Bioanalytical Chemistry* 414 (2022): 6735-6741, https://doi.org/10.1007/s00216-022-04233-x.

[10] Garima. S, Jindal. S, Garg. I. Matai, G. Packirisamy, A. Sachdev, "Dual-Emission Copper Nanoclusters-Based Ratiometric Fluorescent Probe for Intracellular Detection of Hydroxyl and Superoxide Anion Species," *Microchimica Acta.* 188, no. 1 (2021): 13, https://doi.org/10.1007/s00604-020-04683-z.

Figure S10. Stern-Volmer analysis of O-CDs quenched by ·OH at different temperatures.

Figure S11. Effect of NaCl concentration (a-b) and irradiation time (c-d) on the PL intensity of O-CDs.

Figure S12. Cytotoxicity evaluation of O-CDs in Marc-145 cells after 24 h exposure (1.56-100 **μg/mL**).

Figure S13. (a)Temperature-dependent PL spectra of O-CDs in aqueous solution. (b) Comparison of the decrease in PL intensity between the core state and surface state with increasing temperature.
